# Recent Advances in Developmental Hematopoiesis: Diving Deeper With New Technologies

**DOI:** 10.3389/fimmu.2021.790379

**Published:** 2021-11-24

**Authors:** Bart Weijts, Laurent Yvernogeau, Catherine Robin

**Affiliations:** ^1^ Hubrecht Institute-KNAW (Royal Netherlands Academy of Arts and Sciences) & University Medical Center Utrecht, Utrecht, Netherlands; ^2^ Regenerative Medicine Center, University Medical Center Utrecht, Utrecht, Netherlands

**Keywords:** hematopoietic stem cells, aorta-gonad-mesonephros, microenvironment, niche, single cell RNA sequencing, tomography sequencing, embryo, hemogenic endothelium

## Abstract

The journey of a hematopoietic stem cell (HSC) involves the passage through successive anatomical sites where HSCs are in direct contact with their surrounding microenvironment, also known as niche. These spatial and temporal cellular interactions throughout development are required for the acquisition of stem cell properties, and for maintaining the HSC pool through balancing self-renewal, quiescence and lineage commitment. Understanding the context and consequences of these interactions will be imperative for our understanding of HSC biology and will lead to the improvement of *in vitro* production of HSCs for clinical purposes. The aorta-gonad-mesonephros (AGM) region is in this light of particular interest since this is the cradle of HSC emergence during the embryonic development of all vertebrate species. In this review, we will focus on the developmental origin of HSCs and will discuss the novel technological approaches and recent progress made to identify the cellular composition of the HSC supportive niche and the underlying molecular events occurring in the AGM region.

## Introduction

The origin of the hematopoietic system lies within the early developing embryo (1–5). Successive waves give rise to hematopoietic stem and progenitor cells (HSPCs) with various lineage potentials and self-renewal capacities. While initially a pool of short-lived differentiated cells is formed to sustain the fast-growing embryo, multilineage and self-renewing hematopoietic stem cells (HSCs) are then produced to support long-term hematopoiesis. HSCs are first detected in the aorta-gonad-mesonephros (AGM) region prior to colonize mainly the fetal liver (and also the placenta) where they mature and expand through self-renewal. Shortly before birth, HSCs emigrate from the fetal liver and home to the bone marrow (BM) where they form the pool of adult HSCs that will participate to the replenishment of all blood lineages for the remaining life of the organism. For long, stem cells were considered as independent entities able to self-regulate their own behavior. Four decades ago, Schofield was the first to postulate that stem cells are not complete autonomous entities, as they require external signals from the local microenvironment or niche to regulate their behavior and fate decisions to either remain quiescent, to self-renew or to differentiate in response to the need of the organism (6). HSCs are in this regard unique as their formation requires sequential interactions with distinct anatomical sites throughout development and in adults, e.g. the AGM, fetal liver and BM. While HSC niches are well documented in the adult [reviewed in (7–9)], this is far from being the case during ontogeny.

How embryonic and fetal niches exactly support and contribute to the development of HSCs and the hematopoietic system is of great interest both for our fundamental knowledge and for the clinic to advance the therapeutic application of hematopoietic (stem) cells. The increased number of diseases and disorders treated at least in part by HSC transplantations and the difficulties to find HSCs with the best donor-patient compatibility is a major issue. Decades of efforts to develop culture conditions, either to expand HSCs *ex vivo* or to generate new HSCs *in vitro*, hold great promise but success remains limited (10–13). The low production of HSC-like cells with limited multilineage and/or self-renewal properties remains a major barrier to a successful use of HSCs for transplantation and gene modification. The reconstitution of a complete microenvironment or at least some of its key components to support the generation, maintenance and/or expansion of HSCs *in vitro* will be necessary to overcome this barrier. However important clues on how HSC production is supported by successive niches *in vivo* is missing. In this review, we will focus on the developmental origin of HSPCs and will discuss the novel technological approaches and recent progress made to identify the cellular composition, the importance of cell cross-talk and the underlying molecular events involved between the HSCs and the supportive microenvironment in the embryonic aorta, the physiological cradle of the first adult-type HSCs.

## Hematopoietic Production Occurs in Various Highly Vascularized Tissues During Development

In mammals, the first hematopoietic cells are formed independently of HSCs. This first or primitive wave generates nucleated erythrocytes that emerge with and in close proximity to endothelial cells (14). They derive from mesodermal derivatives in blood islands of polyclonal origin, in the extra-embryonic yolk sac (YS), around mouse embryonic day (E)7.25 (15–17). Beside primitive erythrocytes, a first wave of primitive megakaryocyte and macrophage progenitors also arise in the YS blood islands (18–20). A portion of these “primitive” macrophages will persist throughout adulthood and give rise to microglia, the tissue-resident macrophages in the adult brain and central nervous system (21). Multipotent erythro-myeloid progenitors (EMPs) will then produce erythrocytes, macrophages, granulocytes and megakaryocytes (and possibly also few B lymphocytes and NK cells) through a second hematopoietic wave (also referred to as transient definitive wave or EMP wave) in the YS vascular plexus, starting at ∼E8 - E8.25 (18, 22–26). The YS does not provide a competent niche for the differentiation of EMPs in mature cells, which instead occurs in the fetal liver (23). Some macrophages reside in tissues, also referred to as tissue-resident macrophages, of embryos and adults where they act as immune sentinels involved in tissue homeostasis (27, 28). The YS and the developing dorsal aorta in the para-aortic splanchnopleura (which will give rise to the AGM region) also generate lymphoid progenitors at ∼E8.5 - E9.5, independently of HSCs, that are responsible for the initial immunity during development and the persistence of some immune cells (i.e. B-cells) into adulthood (29–33). The importance of these long thought short-life “primitive” hematopoietic cells, of which in fact some subsist and play important roles in late fetuses and adults, reinforces the need for a better identification of the niche-derived signals regulating their production. The 3^rd^ or definitive hematopoietic wave leads to the formation of HSCs that can be detected as early as E10.5, in the aorta of the AGM region and in the extra-embryonic vitelline and umbilical arteries (34–36). Slightly later, HSCs are also found in other highly vascularized tissues such as the YS, and then the placenta and the fetal liver where they expand before colonizing their final destination, the BM (37–40). The impact of the microenvironment on HSC behavior is well illustrated in the fetal liver. During birth, the fetal liver undergoes dramatic changes in hemodynamic forces when the umbilical inlets are ligated. These changes trigger the transformation of arterial endothelial cells lining the portal vessels into venous endothelial cells, characterized by the loss of arterial markers (Neuropilin-1 and Ephrin-B2), acquisition of the venous marker (Eph Receptor B4) and the loss of Nestin^+^NG2^+^ pericytes through apoptosis (41). The latter cells are critical niche components and are probably the main cause of the emigration of HSCs from the fetal liver and their homing to the BM.

Hemogenic endothelial (HE) cells are a small subset of endothelial cells (1–3% in distinct tissues), which have either an arterial or venous identity (28, 42). HE cells can transdifferentiate into hematopoietic cells through a so-called endothelial to hematopoietic transition (EHT), a highly conserved process across vertebrate species (17, 43–49). HE cells can give rise to different types of hematopoietic cells, suggesting that different types of HE cells exist (50, 51). In the floor of the dorsal aorta, HE cells give rise to both EMPs and the first adult-type HSCs (17, 43–47, 49, 52). Most HSPCs are generated from HE cells with arterial characteristics, e.g. in the AGM, vitelline and umbilical vessels and the vascular labyrinth of the placenta and YS (53, 54). However, EMPs can also be produced by venous HE cells in the plexus of the YS (55, 56) and from HE cells in the heart that have not yet acquired an arterial-venous specification (57). Macrophages can also directly derive from HE cells *via* EHT in the placenta (58). The mere presence of HE cells does not guarantee that an EHT event occurs, indicating that the EHT process is differentially steered by signals from the niche, depending on their precise anatomical location and surroundings. For example, HE cells in the aorta that are not exposed to hemodynamic forces from blood flow do not give rise to HSCs, while EMP emergence is unaffected (59–63). How precisely HE cells acquire their hemogenic potential and how this potential leads to the formation of different hematopoietic cells through an EHT event remains to date largely elusive. Nevertheless, there are strong evidences that spatial and temporal signals from the microenvironment play a major role in hemogenic specification (to acquire a hemogenic potential), EHT, IAHC formation and HSPC production. As described in this review, new single cell technologies will shed new light on these processes to better understand how the establishment of the hematopoietic system is regulated.

**Figure 1:**
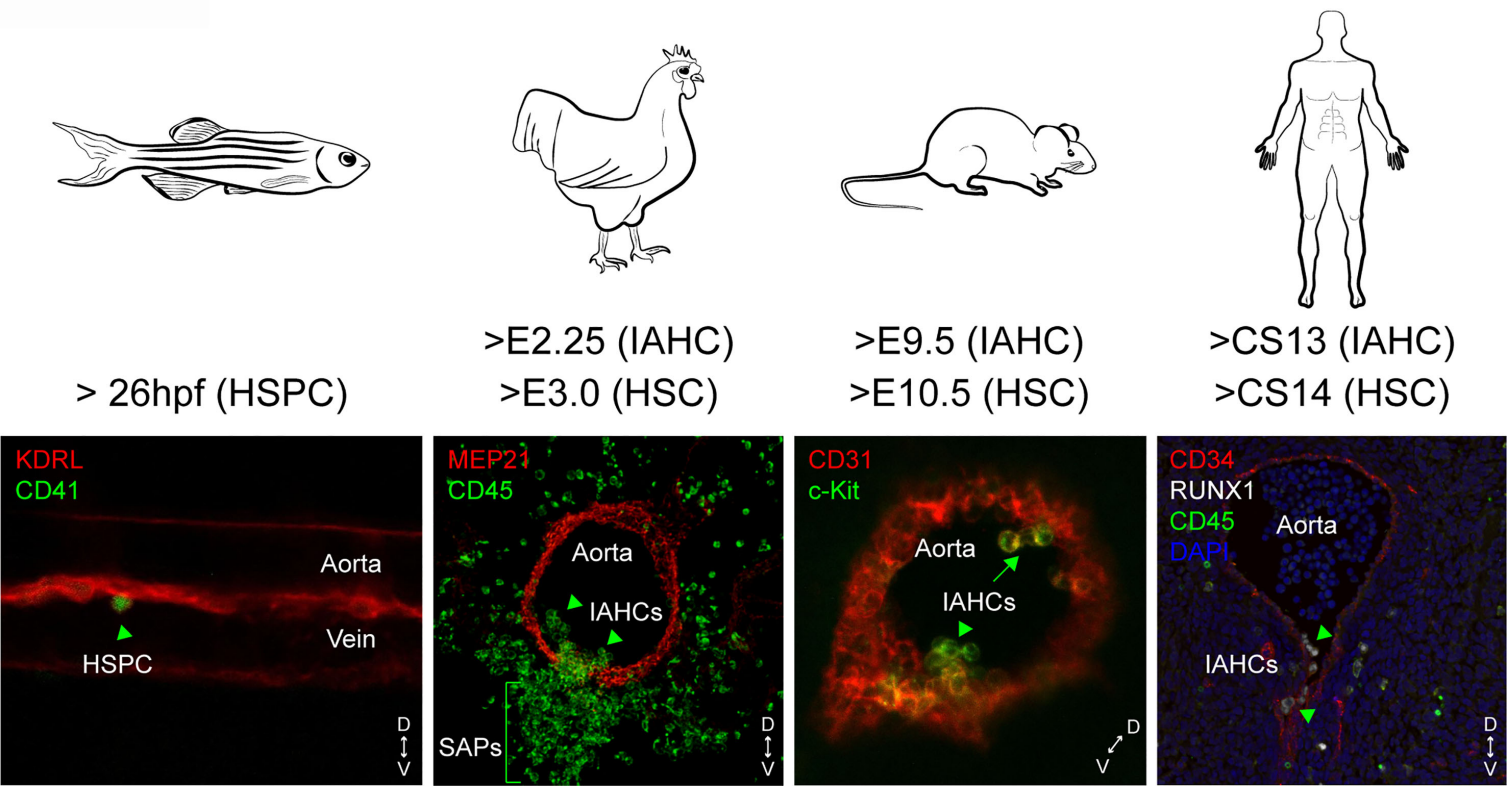
Spatial and temporal locations of IAHCs and HSPCs in the aorta of zebrafish, chicken, mouse and human embryos. The endothelial to hematopoietic transition (EHT) leads to the production of single HSPCs in the aorta of zebrafish embryos, or clusters (intra-aortic hematopoietic clusters or IAHCs). IAHCs emerge exclusively in the ventral side of the aorta in chicken and human embryos (arrow heads). In contrast, IAHCs also emerge in the dorsal side of the aorta in mouse embryos (arrows). In chicken, IAHC cells ingress underneath the ventral aortic endothelium to form sub-aortic patches (SAPs). The starting time of IAHCs and HSPCs/HSCs detected is indicated for each embryo species. D, dorsal; V, ventral.

After EHT, hematopoietic cells are organized in intra-aortic hematopoietic clusters (IAHCs) that remain transiently attached to the inner side of the vessels in most species (44) (**Figure 1**). IAHC-like structures have also been observed in non/less-hematopoietic sites such as the somites or the cerebrovascular sinusoids in the head (64). However, the detection of HSCs (which circulate through the blood circulation) and the presence of IAHCs in a tissue do not prove that EHT occurs *in situ*, as shown in the mouse embryonic head (65, 66). The ultimate proof that the *de novo* formation occurs in a tissue was provided for the aorta by performing confocal imaging on zebrafish embryos *in vivo* and on thick mouse embryo slices *ex vivo* (43, 67–69). The continued existence of HE cells beyond embryonic stages, leading to the formation of multipotent progenitors (MPP-3) and few HSCs in the sinusoids of the BM in fetal/neonate chicken and mouse was also reported and imaged, which could be referred to as a 4^th^ hematopoietic wave (70).

## Spatial and Temporal Emergence of IAHCs and HSC Activity

Extensive research has explored the formation of the first HSCs during development, with a particular focus on IAHCs. IAHC cells express similar hematopoietic and endothelial markers as HSCs (67, 71, 72), and both are absent in *Runx1* knock-out embryos (72, 73), suggesting that HSCs are likely part of IAHCs. However, IAHCs appear earlier than HSCs in the aorta (E9.5 versus E10.5), suggesting that some IAHC cells will become HSCs through gradual specification and maturation (**Figure 1**). Indeed, it was found that IAHCs are mainly composed out of HSC precursors (pre-HSCs type I and type II) that progressively mature into functional HSCs (72, 74–76). Accordingly, IAHCs contain very few HSCs and committed progenitors (that are mainly present in the blood circulation and transiting from the YS to the fetal liver). The process of maturation begins in the aorta but mainly takes place after migration of the cells into the fetal liver and placenta in mammals or in the caudal hematopoietic tissue (CHT) in zebrafish embryos (77). Limiting dilution transplantations and statistical analyses suggest that the pool of adult HSCs in the fetal liver is formed by the pool of IAHC cells (76). This finding raises the question what the contribution of HSCs found in the YS and placenta (39) and the pre-HSCs found in vitelline and umbilical arteries (54) is. A transient production of lympho-myeloid-biased progenitors and lymphoid cells prior to pre-HSC production was also reported in IAHCs (78–80). Although the connections between these different cell types remain unclear, it underlines the heterogeneity and complexity of IAHC composition at different time points and location during development. Of note, various differentiation potentials of IAHC cells have been revealed *ex vivo*, in presence of supportive stromal cell lines (OP-9) and/or cytokines, which might not reflect the true fate of these cells *in vivo*. The EHT is orchestrated by hematopoietic transcription factors primarily driven by RUNX1 (46, 81, 82), although it was reported that *Runx1* deficiency does not preclude the formation of pre-HSCs type I but most likely blocks their maturation into pre-HSCs type II (83). Ectopic expression of Runx1 is sufficient to induce EMPs from non-HE cells (i.e. in YS, aorta, heart), but only between E7.5 to E8.5 of mouse development (82). Therefore, more complex events, such as the initiation of the blood circulation and the proper arterial–venous specialization of endothelial cells in the vascular niche microenvironment, might contribute to temporal restriction of EMPs and (pre-)HSCs appearance in IAHCs.

IAHCs originate from single HE cells that undergo 1 or 2 divisions to form a monoclonal core of IAHCs. Neighboring HE cells are then recruited into IAHCs that become thereby polyclonal while the cellularity increases (84, 85). Intra-cardiac injection of Dll4 blocking antibodies results in the enhanced recruitment of HE cells into IAHCs, suggesting that Dll4-Notch signaling in HE cells regulates IAHC cellularity (84). Unusually large IAHCs were also observed in *Svep1^-/-^* embryos that did not originate from ectopic proliferation of IAHC cells (86). *Svep1* is expressed and secreted by mesenchymal cells surrounding the aorta, highlighting the importance of microenvironmental factors (Notch independent) in determining IAHC size (86). Size and composition might also be determined by an increased proliferation during pre-HSC expansion and a decrease when cells start to acquire HSC identity (87). While slowly cycling cells are located at the base of IAHCs, more proliferating cells preferentially locate at the more apical part of IAHCs (87). Thus, IAHC size is determined by recruitment of neighboring HE cells, controlled by a combination of direct signaling between HE cells and factors derived from the microenvironment, and proliferation of the different cell types within IAHCs. Such local regulation within and between IAHCs is an important concept to further explore since HSC activity is affected by the increased cellularity in IAHCs, as shown in *Svep1^-/-^* embryos (86).

The mechanism of HSC emergence is highly conserved and regulated both in space and time in between species, with few species-specific differences most likely due to anatomical constrains (1) (**Figure 1**). The EHT is polarized and restricted to the ventral side of the aorta in chicken, zebrafish and human embryos (4, 43, 88–91). After EHT, cells form IAHCs that remain transiently attached to the endothelium before detaching and leaving *via* the circulation to colonize the fetal liver in human and mouse. In chicken, the entire floor of the aorta becomes hemogenic, forming IAHC cells that ingress, at least in part, in the mesenchyme underneath the ventral endothelium to form sub-aortic patches, a site considered as the mammalian fetal liver equivalent (89) (**Figure 1**). In zebrafish, HSPCs bud off as single cells from the floor of the dorsal aorta into the sub-aortic space, where they transiently reside and roughly half of them divide before entering the circulation via the posterior cardinal vein (43, 68, 91) (**Figure 1**). Half of these HSPCs are considered as HSCs, the rest being possibly committed myeloid progenitors (92). Although “true” HSCs exist in chicken (89), it remains unknown whether they emerge as pre-HSCs and whether maturation and/or expansion occurs in the sub-aortic patches.

HSPCs start to emerge at 26 hours post fertilization (hpf) with a peak at 40 hpf, in the aorta of zebrafish embryos. IAHCs are found between E2.25 and E5.5 (with a peak at E3) in the anterior portion of the chicken aorta, from days 27 to 42 in the middle portion of the human aorta, and between E9.5 and E14.5 (with a peak at E10.5) in the middle portion of the mouse aorta (4, 34, 43, 68, 88–90, 93, 94). In contrast to other species, the mouse embryo has the particularity to produce IAHCs also in the dorsal part of the aorta (95, 96) (**Figure 1**). RNA-sequencing (RNA-seq) comparative analyses performed on whole IAHCs isolated from the ventral or dorsal part of the aorta revealed a strong similarity at the molecular level at both E10.5 and E11.5 (97). However, dorsal IAHCs are less numerous and have a lower HSC potential (four times less) compared to ventral IAHCs, as shown by limiting dilution transplantations of the subdissected parts of the aorta (ventral [AoV] versus dorsal [AoD]) (95, 98). Using a dissociation-reaggregation culture system that recapitulates HSC development *ex vivo* (99), it was shown that the AoD tissue induces a higher HSC production in the AoV tissue isolated at E10.5 but not at E11.5. In contrast, the AoV induces the production of HSCs in AoD at E11.5 but not at E10.5 (98). Such experiments, although performed *ex vivo*, reveal that the ventral and dorsal aortic microenvironment have reciprocal effects on HSC development, depending of the developmental time, and most likely on the differential release of factors by the two regions (i.e. SCF, Shh, BMP) and the capacity of IAHC cells to respond to specific signals by expressing the right level of receptors at the right time point (75, 98, 100–102). The lack of hemogenic potential of the dorsal aortic endothelium of most other vertebrate species might also be explained by the different origin of the endothelial cells populating the ventral and the dorsal part of the aorta. In zebrafish embryos, the dorsal endothelium does not originate from the splanchnopleura (lateral plate mesoderm) but from the paraxial mesoderm (103). In the avian embryo, the dorsal endothelium that derives from the splanchnopleura is progressively replaced by paraxial mesoderm-derived endothelial cells (as the ventral endothelium), which corresponds to the end of aortic hematopoiesis (104–106).

## Tissue Collections and Transcriptomic Approaches to Unravel the Molecular Landscape of the Aortic Niche

Various interacting signals from unique niche populations present in the different anatomical sites, as well as biomechanical forces, form an intricate signaling network that regulates the formation of HSPCs [for reviews (2, 9, 107–109)]. These signaling events are far from fully understood and many questions remain such as the exact nature (timing and duration) of signaling interactions between the niche and HSPCs and how these interactions contribute to determine different cell fates in endothelial/HE cells and IAHCs. Single-cell (sc) qPCR analyses paved the way for the (single-cell) genomic techniques to explore HSPC development. Although sc-qPCR offers high sensitivity and specificity, the quality of the data relies on high cell numbers and carefully selected and tested primer panels. Such approach identified important players that specify early blood formation (110) or revealed that HE cells are molecularly specified toward a hematopoietic fate two days prior the emergence of the first HSCs (111). Bulk RNA-seq, scRNA-seq and microarray analyses have then been performed to analyze the intrinsic regulation (e.g. by transcription factors) of HSPC formation by sequencing phenotypically enriched populations for arterial endothelial cells, HE cells, cells undergoing EHT, pre-HSCs and/or HSCs sorted from mouse, chicken, human and/or zebrafish embryos (78, 86, 97, 112–123). Overall, these studies highlighted important features. Among them are (i) the molecular heterogeneity of the HE, pre-HSC and HSC populations, (ii) the gene regulatory networks and trajectories involved during HSPC formation, (iii) new surface markers for a better localization and isolation of these rare embryonic cells, (iv) the identification of specific cell-cell-interactions and (v) important novel niche secreted factors. Single-cell transcriptomics of whole mouse and human embryos or organs collected at early time points of development also provided information on early mesoderm specification and important regulators of the early hematopoietic development (124–126). Several signaling molecules and pathways critical for HSC emergence or maturation have been identified by using knock-out/knock-down approaches, large drug screening (in zebrafish) or by performing mouse tissue explant or dissociation-reaggregation cultures in presence of either growth factors or stromal cell lines. Among others are Wnt, Notch, vitamin-A derived retinoic acid signals, BMP4, cytokines such as the interleukin-3 (IL-3) and stem cell factor (SCF), the catecholamines produced by the sympathetic nervous system, pro-inflammatory signals, the blood shear stress, chemokine such as Cxcl12 (SDF1), hyaluronan and extracellular matrix compounds (9, 75, 100, 109, 127–129). Overall, these approaches hardly link a regulator to a specific cell type (e.g. due to the limited purity of the cell populations tested) or to an anatomical location, particularly for the soluble factors.

**Figure 2:**
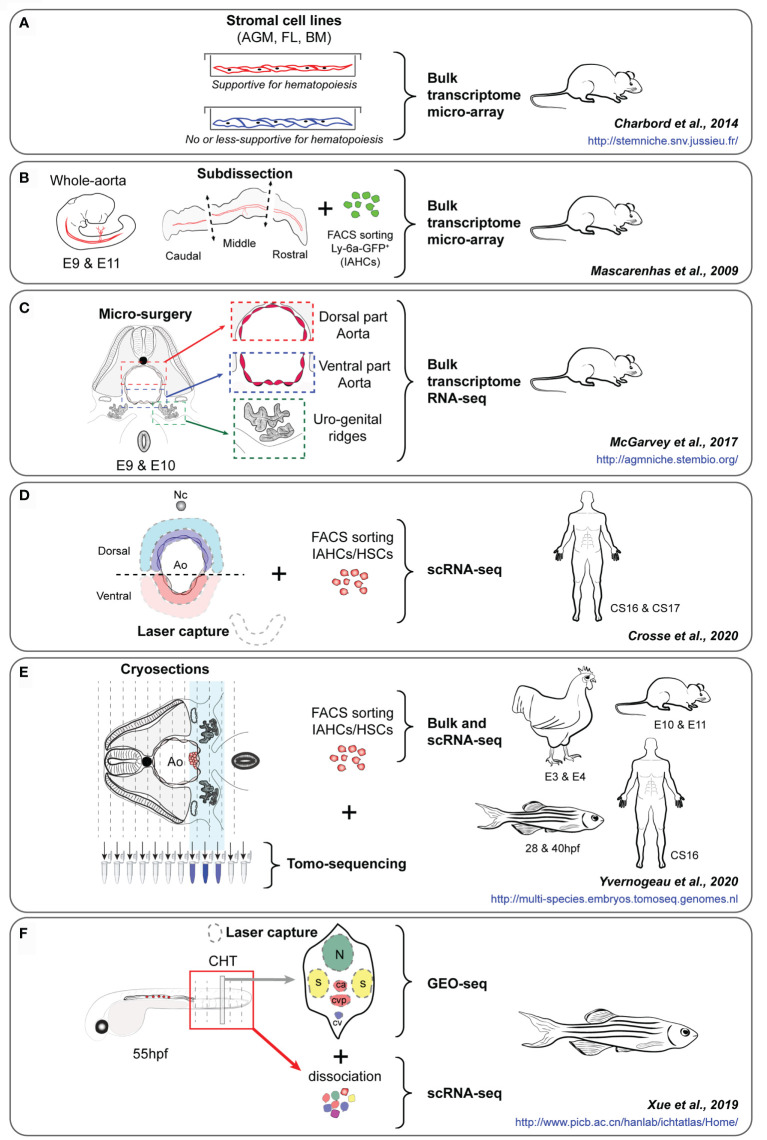
**(A–F)** Experimental approaches of key studies to identify the HSPC supportive landscape of the embryonic aorta, i.e. the studies of Charbord et al. (135) **(A)**, Mascarenhas et al. (93) **(B)**, McGarvey et al. (136) **(C)**, Crosse et al. (117) **(D)**, Yvernogeau et al. (86) **(E)** and Xue et al. (137) **(F)**. The embryo species and stages, the type of cells/tissues analyzed, and the experimental approach used for each study are indicated. The interactives website resources provided in each study are also indicated (in blue). AGM, Aorta-Gonad-Mesonephros; FL, Fetal liver; BM, Bone marrow; E, Embryonic day; IAHCs, Intra-Aortic Hematopoietic Clusters, HSCs, Hematopoietic stem cells; Nc, Notochord; Ao, Aorta; BM, Bone marrow; CS, Carnegie stage; CHT, Caudal hematopoietic tissue; hpf, hour post-fertilization.

In the avian model, dissection procedures that prevent the migration of the sub-aortic mesenchyme, also abolish Runx1 expression in HE cells. Subsequently, the formation of IAHCs is inhibited, proving the supportive role of mesenchymal cells in hematopoiesis in the aorta (130). Notch expression also tightly controls aortic hematopoiesis at specific time points of chicken, zebrafish and mouse development (84, 130, 131). Obtaining a global picture of all the molecular players expressed by the surroundings of the aorta during HSC emergence is still not achieved. In an attempt to identify putative molecules secreted by the HSC supportive microenvironment, several groups have compared cell lines derived from embryonic, fetal and postnatal mouse blood-forming tissues where HSCs emerge, expand or are maintained *in vivo* (e.g. from AGM sub-regions (aorta-mesonephros [AM] and urogenital ridges [UG]), the embryonic liver [EL] or the BM) (132, 133). Such cell lines have been tested and characterized for their competency to maintain/expand mouse and human HSPCs at different levels *in vitro*. Macro-array-based gene expression analyses of HSC-supportive (UG26-1B6 and EL08-1D2) versus less/non-supportive (UG15-1B7, AM20-1B4, EL28-1B3, and AM30-3F4) stromal cell lines revealed an up-regulation of fibroblast growth factor-7 (FGF-7), cathepsin K, thrombospondin 2 (TSP2), pleiotrophin (PTN), and IGFBP-3 and -4 in the supportive cell lines (134). This study demonstrated that ‘niche’ cells are not necessarily in direct contact nor need to be in contact with HSCs to fulfil their support capacity since secreted factors from the microenvironment are sufficient to maintain the HSC stemness properties (134). Using a similar approach, bulk transcriptome comparative analyses of AGM (UG26.1B6 [supportive] *vs* UG26.3B5 [less-supportive]), fetal liver (AFT024 [supportive] *vs* BFC012 [non-supportive]) and BM (BMC9 [supportive] *vs* BMC10 [less-supportive]) cell lines established the genetic signature of the sequential embryonic, fetal and adult HSC niches (135) (**Figure 2A**). Through comprehensive transcriptomic meta-analyses, 481 mRNAs and 17 micro-RNAs were found organized in modular networks and involved in critical signaling pathways. Beside known HSC regulators, this study also identified unexpected ones such as Pax9 and Ccdc80 that were functionally validated using morpholino injections in the zebrafish model. While these studies used solely *in vitro* cell lines, they opened the way for a better identification/characterization of the molecular landscapes of the sequential supportive HSC microenvironments.

Although very informative, studies performed on stromal cell lines that are often clonal do not recapitulate the complexity of the HSC microenvironment. Moreover, critical *in vivo* components (i.e. blood flow/shear stress, circulating cells and growth factors) as well as the spatial three-dimensional organization of the aortic surrounding tissues are missing. It is not certain that all the *in vivo* features need to be reproduced *in vitro*, since HSC development can occur *ex vivo* when AGMs are cultured after dissociation-reaggregation or as explant for few days (35, 99). Several labs explored the aortic microenvironment by dissecting intact hematopoietic organs or by dissecting defined sub-regions, based on their HSC activity (i.e. emergence, expansion). In mice, the middle third of the aorta was identified as the HSC-containing region compared to the most anterior or posterior third regions that were devoid of HSCs (93) (**Figure 2B**). A micro-array transcriptomic analyses performed on these different regions collected at E9 and E11 (before and during the acquisition of an HSC potential, respectively) identified p57Kip2 and IgF2 as important hematopoietic regulators (93). Using a more precise microsurgery, the dorsal and ventral parts of the aorta and the urogenital ridges (UGRs) were isolated from E9.5 to E10.5 since the polarity along the dorsal to ventral axis of the embryo was demonstrated as a clear demarcation of the supportive HSC niche (136) (**Figure 2C**). Bulk RNA-sequencing on these different tissues allowed to identify critical signaling pathways and several secreted molecules, including Bmper as a ventrally polarized new regulator of HSC development in the AGM region. The use of human embryos at early stages when IAHCs appear is challenging due to the difficulty to collect intact embryos at the time of IAHC emergence in the aorta [around Carnegie stage (CS) 13 (post-fertilization age of 30-33 days) (4)] Different layers of cells surrounding the ventral and dorsal sides of the aorta of human embryos were captured by laser dissection and sequenced (LMO-seq) (117) (**Figure 2D**). In parallel, scRNA-seq data obtained from sorted human CD34^+^ IAHC cells were used to explore the cross-talk (e.g. involving secreted factors, cell surface receptors) occurring between IAHCs and the surroundings of the aorta. This approach allowed to highlight the cardiac epidermal growth factor (EGF) and its major receptor, endothelin 1 (expressed and secreted by endothelial cells), as a potent enhancer of HSC generation in human embryos (117).

A major drawback in single cell and bulk sequencing experiments is the lack of spatial information, which is essential to understand the interactions between niche and HSC cells. To perform RNA-seq while keeping spatial information, tomography-sequencing (tomo-seq) was developed (138). Embryonic thick slices or complete embryos are sequentially cryosectioned along the axis of the embryo, e.g. following a transversal or longitudinal orientation, and RNA from each slice is collected for sequencing. RNA expression profiles can then be visualized along the embryo axis. Genes expressed highly or solely in specific tissues/areas can be used for anatomical or orientation confirmation, e.g. *Shh* expression to locate the notochord, or *Mpl* (chicken), *Gata2* (mouse) or *Cxcl12* (zebrafish) expression for the aortic region containing IAHCs/HSPCs (86) (**Figure 2E**). Genes expressed in tissues or regions of interest can then be readily filtered out. By using this technique, thick transversal embryo slices and/or embryo trunks were collected from the 4 main species used to study developmental hematopoiesis, i.e. zebrafish, chicken, mouse and human, at two different developmental time points (beginning and time of HSPC production) (86). Genes specifically expressed in the ventral mesenchyme located underneath the aorta, and more expressed in this region than in the rest of the tissue sample, were identified. To further enhance the usability of these data, transcriptomic datasets of sorted mouse (97) and chicken (86) IAHC cells were generated and compared to the tomo-seq datasets to identify genes and pathways potentially involved in the cross-talk between IAHCs and the ventral aortic microenvironment. Known ligands and corresponding receptors in the aortic ventral microenvironment and IAHC cells were identified, validating this experimental approach and analysis. These were known to be involved in EHT and HSPC survival, attachment, maturation, and/or expansion (e.g. integrin, WNT, BMP, FGF, NOTCH, catecholamines), in inflammation, extracellular matrix organization, cytoskeleton rearrangement, in various cellular processes (protein phosphorylation, intracellular protein transduction) and specific pathways (Pi3K-AKT, MAPK, ERK, RAP1, and RAS). Molecules with unknown hematopoietic role were also identified and functionally validated *in vitro* and *in vivo* as important and conserved HSPC regulators in mouse, chicken and zebrafish embryos. These included (1): the adrenomedullin (ADM), a hypotensive and vasodilator agent and its receptor RAMP2, which regulates HSPC emergence in the aorta, and (2) SVEP1 (Sushi, Von Willebrand Factor Type A, EGF and Pentraxin Domain Containing 1), a secreted extracellular factor critical for proper lymphangiogenesis (139), that was shown to also regulate IAHC cellularity and HSPC production in the aorta (86).

The cellular and molecular mechanisms underlying HSC and multipotent progenitor expansion remain poorly understood. Zebrafish embryos have been used to generate a “3D transcriptional atlas” to characterize the spatiotemporal transcriptome during HSPC expansion in the CHT region (137) (**Figure 2F**). In this study, multi-dimensional RNA-seq approaches were used, including bulk and scRNA-seq on HSPCs isolated at 6 different time points (between 28 hpf and 3 mpf), and *in vivo* GEO-seq performed on the CHT region where six regions were collected on embryo sections by laser capture microdissection at 55 hpf. These regions included the neural tube (in the dorsal region), the muscles (left and right regions), and the caudal artery, caudal vein and caudal vein plexus (in the middle, intermediate and ventral regions, respectively). Such approach combined with functional validation allowed to reconstruct the panoramic transcriptome landscape (temporal and spatial) of the zebrafish CHT, and highlighted the integrin signaling protein Smchd1 as critical for HSPC expansion. Single-cell and spatial transcriptomics recently provided a spatio-temporal transcriptome map of the mouse fetal liver and thereby identified transcriptionally heterogeneous HSPC subsets, as well as HSC ‘pocket-like’ units composed of niche cells (i.e. hepatoblasts, stromal cells, endothelial cells, and macrophages), where macrophages and growth factors (MDK, PTN, and IGFBP5) played an important role in HSPC expansion (140).

## Technological Advances Towards the Molecular and Cellular Dissection of the HSC Microenvironment

**Figure 3:**
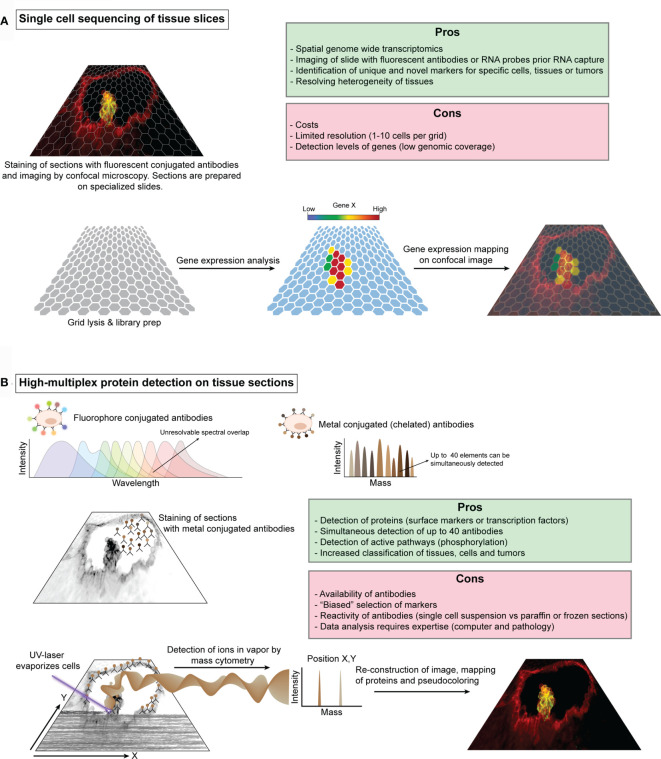
Schematic illustration of single cell sequencing of tissue slices and high-multiplex protein detection on tissue sections. **(A)** Schematic representation of 10X Genomics Visium workflow. Fresh-frozen or paraffin embedded tissue sections are prepared on specialized slides that contain several capture areas or grid. Each grid position has a known unique barcode, which is used for reconstructing the tissue after sequencing. These tissues can be stained with fluorescent antibodies or with one of the principle tissue stains, e.g. hematoxylin & eosin, and subsequently imaged by microscopy prior RNA capture. Cells are then permeabilized and the RNA content is captured for barcoding and library preparation. The library is sequenced by standard procedures and the data can be visualized in relation to the imaged tissues. **(B)**. The use of multiple fluorescent proteins and quantum dots to simultaneously label different cell components is limited to the ability to resolve spectral overlap between the different fluorochromes. To increase the number of labels, classic fluorescent proteins have been replaced by specific elements or isotopes such as noble and post-transition metals, rare-earth elements and halogens, which can be detected by mass cytometry. Antibodies against specific proteins (e.g. receptors or transcription factors) can be tagged with these isotopes and used for staining of tissue sections and an image of the general morphology can be taken. A UV-laser evaporates the cells in a typical confocal scanning movement (X,Y) and fumes of the cells are transported by an inert gas into the mass cytometer for measurement. The measured isotopes and the corresponding X,Y coordinates can then be used to reconstruct an image which can be superimposed on top of the microscope images.

The ability to visualize HSCs in their native environment has been paramount for our current knowledge regarding HSC dynamics and behavior *in vivo* (43, 48, 67, 68, 89, 141, 142). However, immunostainings, *in situ* hybridizations and the use of transgenic reporters only allow for the simultaneous visualization of a handful of genes. Ideally, one would like to image HSCs embedded in their niche in detail, or even follow them by time-lapse imaging until they display the desired behavior, capture the entire transcriptome and map this back to the imaging data. This combination would permit the precise identification of the different (sub)types of cells, as well as their transcriptomic state. Such approach would be especially powerful to e.g. elucidate the exact composition of small and large IAHCs, the heterogeneity of various endothelium (hemogenic and non-hemogenic) and the direct interactions of HSCs with their successive embryonic, fetal and adult niches. Although laser micro-dissection allows for the isolation of relatively small sections of tissue after imaging that can be processed for transcriptomic analysis (137), current technological advances are heading towards a more precise capture of the transcriptome of a full slide. Visium spatial gene expression by 10X Genomics permits such capture of the whole transcriptome from a tissue section. Prior capture, there is an optional step that allows for the visualization of proteins of interest by immunofluorescence to gain a deeper understanding of tissue organization or the localization of cells of interest (**Figure 3A**). This first version of Visium has 5.000 uniquely barcoded spots on 4 separate capture areas on each slide. Each spot will capture a range of about 1-10 cells, depending on the tissue thickness and tissue architecture (e.g. cell size). This capture grid will undoubtedly become smaller in future versions to reduce the number of cells captured per spot. While such approach will definitely add to our understanding of how HSCs are embedded and interacting with their microenvironment, the financial burden to systematically study HSCs in their native environment will be extremely high.

Whilst much attention goes out to transcriptomic approaches (122), recent advances in protein-based techniques should not be overlooked. Measuring proteins present in or on the cell by fluorescence-based flow cytometry has proven to be a rapid and powerful tool for isolating, sub-typing and phenotyping the cells of the immune system, including HSCs (143, 144). Multiplexing classic fluorescent and quantum dots labeled antibodies have stretched the limit of this technique up to 17 parameters (145, 146). Further expansion of fluorescent based cytometry seems unlikely due to the limitations to resolve the spectral overlap. Replacing fluorescent proteins or quantum dots with element isotopes (chelated antibody tags) dramatically reduced the cross-talk between channels and enabled the simultaneous measurement of up to 40 parameters, which is referred to as cytometry by time-of-flight (CyTOF) (147, 148). Distinct isotopes can be used to label different antibody panels that include surface markers, transcription factors as well as signaling molecules (phosphoproteins). By using CyTOF with about 31 different isotopes, functional and hierarchical maps of the immune/hematopoietic system have been drawn and show that hematopoiesis is a continuum rather than a collection of defined subsets (149–151). CyTOF was also instrumental in identifying a pro-inflammatory subset of macrophages that is involved in the development of HSCs (152). In addition, integrating CyTOF with scRNA-seq could provide additional discriminatory power for further sub-setting or functional analysis between distinct subsets of cells like HSCs and progenitors (153). Besides the precise immunophenotyping of cells in suspension, recent advances have enabled CyTOF of tissue sections or cells cultured on a slide. Aerosols of evaporated cells by laser ablation are transported to the CyTOF mass cytometry by an inert gas for detection (154). Data for each cell is then mapped based on the laser ablation coordinates to reassemble the original tissue architecture (**Figure 3B**). While the selection and availability of antibodies used is crucial to the success of this technique, it provides a new powerful way to study for example sections of the embryonic aorta to elucidate the composition of the vascular aortic, fetal liver and BM niches.

## Recapitulating the Endogenous HSC Niche in 3D-Culture Systems to Produce *bona fide* HSCs and Other Blood Forming Cells

Limitless access to different types of blood producing cells manufactured *in vitro* is the “holy grail” of regenerative medicine. This would, among others, combat the current shortage of donor HSCs by providing a readily accessible source to all blood groups (red blood cells) (155, 156) or functional T-cells that can be engineered for anti-cancer therapies (157, 158). The crux of the matter is that these *in vitro* produced cells should faithfully mimic their *in vivo* counterparts. *In vitro* production of hematopoietic progenitors and mature blood cells (e.g. red blood cells, platelets, megakaryocytes, T-cells) from pluripotent stem cells or somatic cells, through reprogramming or transgene free protocols, is achievable (159). Reprogramming by (transient) expression of transcription factors is also a very promising strategy to generate HSC-like cells *in vitro* since a decade (11, 160–166). However, these HSC-like cells are produced at a very low yield and remain limited in their capacities to self-renew and/or to replenish all blood lineages, which is an absolute requirement for therapeutic use. Moreover, the association of the reprogramming factors with the development of leukemia remains an underlying risk. Indirect reprogramming (transgene free) through co-culturing pluripotent stem or progenitor cells with supportive cells that mimic the microenvironment in combination with chemical manipulation has therefore become a more favorable option. In its simplest form, this would be co-culturing pluripotent stem cells (e.g. iPSCs, ESCs) or somatic cells with a supportive cell line (e.g. OP9-cells) and/or a cocktail of growth factors, hormones and/or cytokines. Under these conditions, the formation of HE cells and some hematopoietic progenitors was successfully obtained, while HSCs are not or very rarely produced (167–173). As discussed above, the formation of HSCs *in vivo* requires a chain of events involving a complex sequence of both cell intrinsic and extrinsic factors that are difficult to recapitulate in a “simple” *in vitro* setting. Furthermore, one of the important open standing questions is whether it is necessary to first mimic the AGM-like microenvironment, to ensure HE formation and pre-HSC production *in vitro*, and second to mimic the fetal liver microenvironment to support pre-HSC maturation and HSC expansion. Comparing the transcriptomes of *in vitro*-generated HSC-like cells to fetal liver HSCs is an interesting approach to identify transcription factors and molecular pathways that could improve the *in vitro* production of HSCs (174). However, *in vitro*-generated HSCs that faithfully mimic the functionality of *bona fide* HSCs might have a different transcriptional landscape.

Recent advances in the *ex utero* culture of post-implantation mouse embryos, enabling the development until the hindlimb formation stage (E11) (175), or in 3D-culturing systems (176, 177) might offer a more sophisticated way of producing transplantable HSCs *in vitro*, as these systems recapitulate key aspects of developmental processes or organs. Disaggregation-reaggregation assays in the 1950’s showed that a suspension of chicken mesonephric cells could self-organize into the structural pattern of the original tissue, now generally referred to as organoids (178, 179). To date, organoid technology is mainly used to model the development of organs “in a dish”, including various diseases affecting these organs. The AGM is a complex region composed of a myriad of cells, including endothelial cells, HE cells, mesenchymal cells and various immune cells and it would be a challenging task to mimic the precise organization and timing of influx of supportive immune cells, like macrophages, within organoids. Hence, reports of successful HSC production by organoid technology are scarce, although there are some 3D iPSC-derived organoid-like culture systems that produce hematopoietic progenitors (180). The succeeding step of organoid cultures are techniques to mimic the first days of embryonic development in a dish. Pluripotent stem cells or iPSCs in a round bottom plate or in a hanging drop will aggregate into embryoid bodies (EBs) to form the three embryonic germ layers. Providing these EBs with a tailored cocktail of growth factors and cytokines at the right time will result in the production of endothelial cells, hematopoietic progenitors, erythrocytes, macrophages, neutrophils and mast cells (181–186). Until recently, this approach did not yield any transplantable HSCs. However, by extending the EB culture and optimizing the cocktail of cytokines and growth factors, which includes BMP4, VEGF, IGF1, SCF, FLT3, TPO, IL-1, -3, -6 and G-CSF, the formation of multilineage HSCs that produced myeloid, lymphoid and erythrocytes in sequential transplanted recipients was achieved (187). The success of generating HSCs from EBs might result from the extension of the EB culture time, allowing germ-layer specification and lineage commitment, or alternatively from the maturation of pre-HSCs. Reconstitution of sub-lethally irradiated recipients by these EB-derived HSCs required the injection of 400.000 cells from dissociated EBs per mouse, indicating that the production of HSCs in EBs is extremely low. One potential explanation for the low yield might be the restricted number of cells receiving the required spatial and temporal signals. This might be due to a sub-optimal organization of the different germ layers in these EBs. Interestingly, stimulating EBs with a pulse of a WNT/β-catenin signaling agonist results in the break of symmetry and subsequently leads to an anteroposterior axial organization with a bilateral symmetry similar to vertebrate embryos (188, 189). These so-called gastruloids display key features of mammalian development after implantation although tissue organization is often limited. Improvements in culturing conditions, such as embedding in Matrigel or fusion of a pulsed and non-pulsed EB, enhanced tissue organization and led to the formation of somite-like and neural structures (190–192). Single cell transcriptomics of gastruloids of different “developmental stages” and culturing methods showed different mesodermal derived populations with expression of endothelial and early blood markers like *Etv2, Kdr, Cdh5, Kit, Gata2, Runx1, Cd34* and *Itga2b* (190, 191, 193). This suggests that gastruloids produce a hemangioblast-like cell type that might differentiate into endothelial, HE and/or even early HSPCs. Conformingly, detailed analysis of endothelial and blood markers in gastruloids revealed the formation of a vascular plexus, the production of blood progenitors and erythroid-like populations (193). The presence of blood vessel like structures and expression of markers like *Kit, Itga2b* and *Runx1* is suggestive for the presence of HE cells and/or pre-HSCs, although additional functional assays are needed to confirm whether gastruloids can produce such cell types. Gastruloids are thus a promising and exciting new tool that does not need to use animals (respect of the 3R rules) and might proof valuable in understanding how extrinsic signals derived from the microenvironment instruct HE cells to undergo EHT and maybe produce (pre-)HSCs. However, the limited time when gastruloids can be cultured (equivalent to a mouse embryo around E8.5-E9.5) might preclude the formation of (pre-)HSCs.

## Concluding Remarks

An increasing number of studies based on RNA-sequencing and/or spatial transcriptomics performed in different embryo species confirmed the complexity of the aortic niche. The development of new molecular approaches and the increasing power of scRNA-seq technologies now offers the possibility to go one step further in the study of HSC regulation by the surrounding microenvironment and to generate high throughput datasets with limited material. The main challenge will be to manage and integrate all RNA-seq, spatial transcriptomic datasets in a comprehensive manner to obtain a global/real picture of what is happening in the AGM region when pre-HSCs/HSCs are generated, and to identify the fine tuning of all the regulators that evolve both in time and space. Most datasets are freely accessible and several labs have invested in creating interactive websites, which makes the exploration of these data relatively easy and allows to interrogate for the expression of any mRNA and miR of interest in supportive/non-supportive cell lines (135), gene expression in different sub-dissected regions of the AGM in mouse (136) and in multiple species for comparison and conservation (86), as well as in the CHT, the HSPC expansion niche in zebrafish embryos (137) (**Figure 2**). The number of molecules and pathways identified to be involved in hemogenic specification, EHT, IAHC formation and pre-HSC maturation within the aorta are continuously increasing. However, the mechanism by which most of these molecules interact and/or interfere, directly or indirectly, to regulate HSPC fate remains to be elucidated.

The evolution and combination of sc-genomic and multiomic techniques (e.g. Scifi-seq, ASAP-seq, ECCITE-seq, Visium) and the efforts made to increase cell throughput with lower costs or the detection of rare cell populations will continue to pave the way for a better understanding of HSPC production and its fine-tuned regulation by the supportive niche. The integration of transcriptomics, proteomics, and epigenetic changes at single-cell resolution and functional validations *in vitro* or *in vivo* will be essential to understand HSPC development in physiological condition with the goal to improve cell-replacement therapy, but also in immune and blood disease conditions, e.g. in the case of childhood leukemia that originate *in utero*.

## Author Contributions

BW and LY contributed equally to the review. All authors contributed to the article and approved the submitted version.

## Funding

This work was supported by a European Research Council Grant (ERC, project number 220-H75001EU/HSCOrigin-309361), a TOP subsidy from NWO/ZonMw (912.15.017), a KWF grant (Dutch Cancer Society, project 13549) and the UMC Utrecht “Regenerative Medicine & Stem Cells” priority research program.

## Conflict of Interest

The authors declare that the research was conducted in the absence of any commercial or financial relationships that could be construed as a potential conflict of interest.

## Publisher’s Note

All claims expressed in this article are solely those of the authors and do not necessarily represent those of their affiliated organizations, or those of the publisher, the editors and the reviewers. Any product that may be evaluated in this article, or claim that may be made by its manufacturer, is not guaranteed or endorsed by the publisher.
